# Effect of Overexpression of *JERFs* on Intracellular K^+^/Na^+^ Balance in Transgenic Poplar (*Populus alba* × *P. berolinensis*) Under Salt Stress

**DOI:** 10.3389/fpls.2020.01192

**Published:** 2020-08-14

**Authors:** Changjun Ding, Weixi Zhang, Dan Li, Yufeng Dong, Junlong Liu, Qinjun Huang, Xiaohua Su

**Affiliations:** ^1^State Key Laboratory of Tree Genetics and Breeding, Research Institute of Forestry, Chinese Academy of Forestry, Beijing, China; ^2^Key Laboratory of Tree Breeding and Cultivation of State Forestry Administration, Research Institute of Forestry, Chinese Academy of Forestry, Beijing, China; ^3^Shandong Provincial Key Laboratory of Forest Tree Genetic Improvement, Shandong Academy of Forestry, Jinan, China; ^4^Industry of Timber and Bamboo, Anhui Academy of Forestry, Hefei, China

**Keywords:** poplar, *JERF36s*, ion balance, *NHX1*, *SOS1*, salt resistance

## Abstract

Salt stress is one of the main factors that affect both growth and development of plants. Maintaining K^+^/Na^+^ balance in the cytoplasm is important for metabolism as well as salt resistance in plants. In the present study, we monitored the growth (height and diameter) of transgenic *Populus alba* × *P. berolinensis* trees (ABJ01) carrying *JERF36s* gene (a tomato jasmonic/ethylene responsive factors gene) over 4 years, which showed faster growth and significant salt tolerance compared with non-transgenic poplar trees (9#). The expression of *NHX1* and *SOS1* genes that encode Na^+^/H^+^ antiporters in the vacuole and plasma membranes was measured in leaves under NaCl stress. Non-invasive micro-test techniques (NMT) were used to analyse ion flux of Na^+^, K^+^, and H^+^ in the root tip of seedlings under treatment with100 mM NaCl for 7, 15, and 30 days. Results showed that the expression of *NHX1* and *SOS1* was much higher in ABJ01 compared with 9#, and the Na^+^ efflux and H^+^ influx fluxes of root were remarkable higher in ABJ01 than in 9#, but K^+^ efflux exhibited lower level. All above suggest that salt stress induces *NHX1* and *SOS1* to a greater expression level in ABJ01, resulting in the accumulation of Na^+^/H^+^ antiporter to better maintain K^+^/Na^+^ balance in the cytoplasm of this enhanced salt resistant variety. This may help us to better understand the mechanism of transgenic poplars with improving salt tolerance by overexpressing *JERF36s* and could provide a basis for future breeding programs aimed at improving salt resistance in transgenic poplar.

## Introduction

The total area of arid, semiarid and saline-alkali lands in China accounts for more than half of the total terrestrial with low underground water levels and serious salinization. Among them, soil salinization is a major abiotic stress, and approximately 7.6 million hm^2^ of farmland (almost 1/5 of the total) shows certain degrees of salinization (Song et al., 2016; Ke et al., 2017; Chen et al., 2019). Salt stress has various adverse effects in plant growth and development, including osmotic stress, ion toxicity, water shortage, and nutrient imbalances (Zhu, 2001; Kaleem et al., 2018; Liang et al., 2018; Yang and Guo, 2018; Chen et al., 2019). The prevalence and impact of high salinity on plant growth and productivity have drawn increasing attention (Widodo et al., 2009; Zhu et al., 2016; Yang and Guo, 2018). *Populus* is an economically and ecologically important perennial woody plant that is widely cultivated in the Northern Hemisphere because of their rapid growth and high biomass yields, with marked economic benefits and applications in the production of biofuels, pulp, paper, and other bio-based products, such as chemicals and adhesives (Sannigrahi et al., 2010; Meng and Wu, 2018; Zhang X. N. et al., 2019). However, soil salinization is an increasingly serious threat that limits poplar growth and afforestation program, so breeding salt-tolerant poplar has become very important and necessary.

The application of genetic engineering and transformation technology provides effective means of improving salt tolerance of poplar by introducing known salt-responsive genes into *Populus* (Jansson and Douglas, 2007; Li et al., 2009; Su et al., 2011; Ye et al., 2011; Zhou et al., 2014; Yao et al., 2017; Zhang X. N. et al., 2019). Many studies have shown that inroducing transcription factors (TFs) genes into plants is generally more efficient than structural genes, because TFs usually regulate the expression of many target genes in related pathways, leading to enhanced stress torlance in plants (Mcgrath et al., 2005; Li et al., 2009; Su et al., 2011; Licausi et al., 2013; Karim et al., 2015; Yao et al., 2016; Wei et al., 2017; Zhao H. et al., 2018; Charfeddine et al., 2019; Zhang C. H et al., 2019; Zhang X. N. et al., 2019; Chen et al., 2020; Jiang et al., 2020). Ethylene responsive factors (ERFs), which have a characteristic AP2/ERF DNA-binding domain, comprise one of the largest TF families in plants (Riechmann et al., 2000; Nakano et al., 2006; Licausi et al., 2013; Zhang X. N. et al., 2019; Chen et al., 2020). Overexpression of ERFs genes can effectively improve salt tolarence of plant under salt stress, such as *ERF96* (in *Arabidopsis thaliana*) (Jiang et al., 2020), *StERF94* (in potato) (Charfeddine et al., 2019), *ERF76* (in di-haploid *P. simonii* × *P. nigra*) (Yao et al., 2016; Yao et al., 2017; Yao et al., 2019), *PalERF109* (in *P. alba* var. *pyramidalis*) (Chen et al., 2020), and *JERFs* (in tomato) (Li et al., 2009; Su et al., 2011).

*Populus alba* × *P. berolinensis* exhibits fast growth and beautiful tree shape, and is widely used in urban greening and afforestation in northeast, northwest and north of China. However, *P. alba* × *P. berolinensis* is difficult to plant on salinity soil because of its susceptibility to salt stress, which seriously affects its popularizing application on Saline-alkali land. To improve salt resistance in this variety, a tomato jasmonic ethylene responsive factors (*JERF36s*) gene that encodes the ERF like transcription factor (Zhang and Huang, 2010) has been transferred to the genome of the *P. alba* × *P. berolinensis* in our previous study (Li et al., 2009). Overexpression of *JERF36s* resulted in the improved salt tolerance in transgenic poplar (ABJ01) compared with non-transgenic poplar (9#) (Li et al., 2009). However, how *JERFs* exactly improve salt resistance in transgenic poplar remains to be unravelled.

The maintenance of intracellular K^+^ and Na^+^ homeostasis is considered to be an essential component for plant growth or adaption in saline environments (Munns and Tester, 2008; Huang et al., 2018), which is a crucial factor affecting plant salinity tolerance (Chen et al., 2007; Huang et al., 2018; Zhang X. N. et al., 2019; Blumwald et al., 2000). Maintaining a cytosolic K^+^/Na^+^ homeostasis balance largely depends on preventing the accumulation of Na^+^ *via* Na^+^ expulsion from cell cytoplasm, sequestering Na^+^ into vacuoles, and increasing K^+^ absorption (Hasegawa, 2013; Bassil and Blumwald et al., 2014; Yang and Guo, 2018; Bassil et al., 2019; Su et al., 2019). The Na^+^, K^+^/H^+^ antiporters are the transmembrane protein locating in both plasma and vacuolar membranes (Guo et al., 2020). Na^+^, K^+^/H^+^ antiporter (NHX)-type exchangers are the critical regulators of vesicular tracking and cell volume, which catalyse the electroneutral exchange of K^+^ or Na^+^ for H^+^ and play vital roles in plant salt tolerance, as well as the Na^+^ and K^+^ homeostases of intracellular compartments (Bassil and Blumwald et al., 2014; Azhar et al., 2017). AtNHX1 and AtNHX2 are vacuolar-type NHXs and localized to the vacuolar membrane, which play diverse roles in maintaining Na^+^/K^+^ homeostasis under salt-stress (Leidi et al., 2010; Bassil et al., 2011), as well as in active K^+^ uptake in the tonoplast for stomatal function (Barragán et al., 2012). Another plasma membrane localized Na^+^/H^+^ exchange transporter SOS1 can be activated by SOS3-SOS2 protein kinase complex under high Na^+^ stress, resulting in the exclusion of excess Na^+^ from plant cells, which is essential for maintaining Na^+^ and K^+^ homeostasis in the cytoplasm and defense against salt stress (Zhu, 2002; Qiu et al., 2004; Hu et al., 2016; Zhu, 2016). *SOS1*, belongs to the NHX-type Na^+^(K^+^)/H^+^ exchange family genes (*NHXs*). Some studies have described that overexpression of *NHXs*, such as *NHX1* and *SOS1*, can effectively maintain Na^+^/K^+^ homeostasis in the plant cells for the enhancement of salt tolerance (Wu, 1996; Shi et al., 2000; Barragán et al., 2012; Ma et al., 2014; Li et al., 2017; Fahmideh and Fooladvand, 2018; Huang et al., 2018; Liang et al., 2018; Meng and Wu, 2018; Wang et al., 2018; Acuña-Rodríguez et al., 2019; Liu et al., 2019; Mahi et al., 2019; Yao et al., 2019; Zhao et al., 2019). However, there are limited reports of the regulation of intracellular K^+^/Na^+^ balance by TFs in transgenic poplar with exogenous transcription factors.

In the present study, to explore the effects of *JERFs* on intracellular K^+^/Na^+^ balance, the transgenic poplar overexpressing *JERF36s* (ABJ01) and non-transgenic poplar (9#) as control were grown in field trials, and the expression of *NHX1*(Gene ID: 105132046) and *SOS1*(Gene ID: 7462643) genes, encoding Na^+^/H^+^ reverse transporters in the vacuole membrane and plasma membrane was measured. Non-invasive micro-test techniques (NMT) were used to analyse the ion flux of Na^+^, K^+^, and H^+^ in the root tip of seedlings stressed by 100 mM NaCl for 7, 15, and 30 days. These findings may provide a basis for future breeding programs aimed at improving salt resistance in transgenic poplar.

## Materials and Methods

### Materials

The hybrid poplar from *Populus alba* × *P. berolinensis* (9#) was used in this study. Transgenic poplar line (ABJ01) were produced as described previously (Li et al., 2009). Briefly, The transfected exogenous gene was the *JERF36s* gene, encoding AP2/EREBP plant transcription factors, which was under the control of the CaMV 35S promoter. The neomycin phosphotransferase II gene (NPT II) derived from Escherichia coli transposon Tn5 was used as a marker, which provided the plants with kanamycin resistance. The 4-year study plots of 9# and ABJ01 were established in 2004 in a forest located in Panjin, Liaoning Provence (41°170' N, 121°360' E) at an elevation of 14 m above sea level. The soil salt content was measured before the forest was established. Soil samples were taken from 0–10 cm, 10–30 cm, and 30–50 cm depths in three randomly chosen places. The average salt content and pH at the different depths were as follows; 0–10 cm = 0.38% and pH 7.30; 10–30 cm = 0.2% and pH 7.40; 30–50 cm = 0.18% and pH 7.50. The average total salt content was 0.25%. Established trees (1-year-old) from 9# and ABJ01 were planted in a randomized block design. The field trial consisted of four blocks, each containing three replicates for each line. The forest was planted in 2 m rows with 1 m between adjacent individuals within each row. Cuttings (15 cm) from annual seedlings of 9# and ABJ01 were used for ion flux measurements and qRT-PCR. Cuttings (15 cm) from annual seedlings of 9# and ABJ01 were placed in individual plastic pots of 10 cm in diameter and 10 cm in depth on the 15^th^ of March 2014 and cultured in a greenhouse at the Chinese Academy of Forestry in Beijing, each line was 15 plants. The stroma in pots was a mixture of peat soil and perlite. On 21, April, 9 healthy and similar growth plants of each line were selected to transplant into larger pots (19 cm in height and 17 cm in diameter) in a mixture of sandy soil, peat soil, and pearlite at a ratio of 6:10:1 with 1.8 kg of soil per pot, which were used to measure the growth, ion flux and gene expression in the further. Seedlings were watered regularly, the temperature of the greenhouse was maintained between 25°C and 30°C, and the humidity was 55%.

### Salt Treatment and Measurement of Growth

18 seedlings from each of the 9# and ABJ01 groups were selected when the height of the seedlings reached 40 cm. Nine seedlings from each group were set aside as controls, and nine were treated with 100 mM NaCl solution every 2 days for 7, 15, or 30 days with three triplicates, respectively. The growth was determined by measuring the height of each plant after 30 days of salt stress.

### Measurement of Height and Diameter in the Field

Growth measurements were completed in 2004, 2005, 2006, and 2007. The height (the distance from a point at ground level to the top of the tree) and diameter at breast height (DBH, 1.3 m above the ground level) of ABJ01 and 9# were measured each year after the growing season, each line was measured in 12 plants as replicates.

### Expression of *JERF36s*, *NHX1*, and *SOS1* Analyses by qRT-PCR

One or two leaves from the top of seedlings were harvested after treatment with 100 mM NaCl for 7, 15, or 30 days. Harvested leaves were frozen in liquid nitrogen immediately and stored at -80°C until needed for RNA extraction. Total RNA was isolated from leaves using a RNeasy Plant Mini Kit (Qiagen) according to the manufacturer's instructions. cDNA was synthesized using the PrimerScript RT reagent Kit (TaKaRa, Dalian, China). Quantitative RT-PCR (qRT-PCR) was performed on a Roche LightCycle 480 Real-Time PCR system (Roche) with SYBR Green Realtime PCR Master Mix (TaKaRa) according to the manufacturer's instructions. Gene-specific primers were designed to amplify 120–130 bp fragments of *JERFs*, *NHX1*, and *SOS1*, and the relative gene expression were calculated by 2^-ΔΔCt^ methods using a gene-specific primer pair for poplar ubiquitin (UBQ)-like gene (GenBank Accession BU871588) as a reference gene (Livak and Schmittgen, 2001). Primer sequences for the real-time PCR assay of the genes are listed in **Supplementary Table S1**. Six plants for each line were tested, and four PCR replicates were performed for each RNA sample.

### NMT Technique for Measuring Na^+^, K^+^, and H^+^ Ion Flux

The net Na^+^, K^+^, and H^+^ ion flux was measured using an NMT system (BIO-001A3, Younger USA Science & Technology Corporation, USA) (Xu et al., 2006; Xu et al., 2008; Sun et al., 2009a; Sun et al., 2009b; Yan et al., 2015a; Yan et al., 2015b; Liang et al., 2018; Zhao N. et al., 2018). The NMT system measured the concentration gradients of the target ions by moving the ion-selective microelectrode between two positions close to the plant material through a pre-set excursion distance (30 mm in our experiment) at a programmable frequency of 0.15 Hz (Sun et al., 2009a; Zhao N. et al., 2018). Ion-selective electrodes were made as described previously by Sun (2009a) and Yan (2015a; 2015b). Ion-selective electrodes were calibrated in calibration solutions (0.5 mM Na^+^, 5.0 mM NaCl; 0.1 mM K^+^, 1.0 mM KCl; H^+^, pH 5.5, and 6.5) prior to flux measurements (Sun et al., 2009a; Sun et al., 2009b; Yan et al., 2015a; Yan et al., 2015b; Zhao N. et al., 2018). Only electrodes with Nernstian slopes between 53 and 62 mV/[log-C (± z)] were used in our study (where z is the valence of the ion and C is the concentration of the ion) (Yan et al., 2015b). Ion flux was calculated by Fick's law of diffusion:

J=−D(dc/dx)

where J represents the ion flux, dc/dx is the ion concentration gradient, and D is the ion diffusion constant in a particular medium (Sun et al., 2009a; Sun et al., 2009b; Yan et al., 2015a; Yan et al., 2015b).

Fresh and tender roots from seedlings were treated with salt for 7, 15 or 30 days, and 20 mm from the root tip was removed from 2–3 seedlings in each group (performed in triplicate) for ion flux measurements. Washed root tips were placed in test solution for 25–30 min prior to measurement, then transferred to 10 mL of test solution in a transparent culture dish and fixed to the bottom of the dish. Ion-selective electrodes were passed through the root at 600 μm, 1,200 μm, and 1,800 μm from the root tip, and each measurement was made for at least 5 min to ensure a stable ion velocity. A positive value indicates external ion flux, and a negative value indicates internal ion flux.

### Statistical Analyses

One-way analysis of variance (ANOVA, = 0.05) was used to analysis the differences between the ABJ01 and 9# for growth, qRT-PCR and, Na^+^, K^+^, and H^+^ ion flux, and multiple comparison of means was done using Duncan's multiple range test. All analyses were done with Data Processing System (DPS) software (Tang and Feng, 2002).

## RESULTS

### Growth of 9# and ABJ01 Under Salt Stress

**Figure 1:**
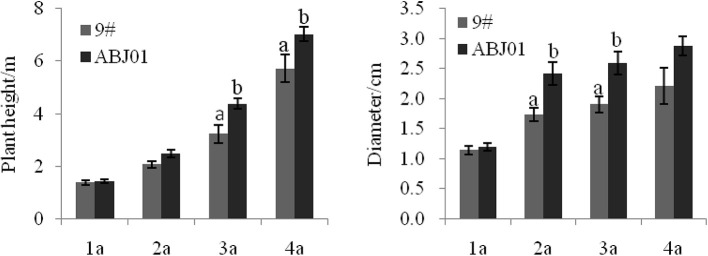
The growth (height and diameter) of 9# and ABJ01 over a 4-year period (2004–2007). The data shows mean ± S.E. of three blocks. Column with different letters indicate significant differences from control (9#) (p < 0.05). N = 12 plants.

**Figure 2:**
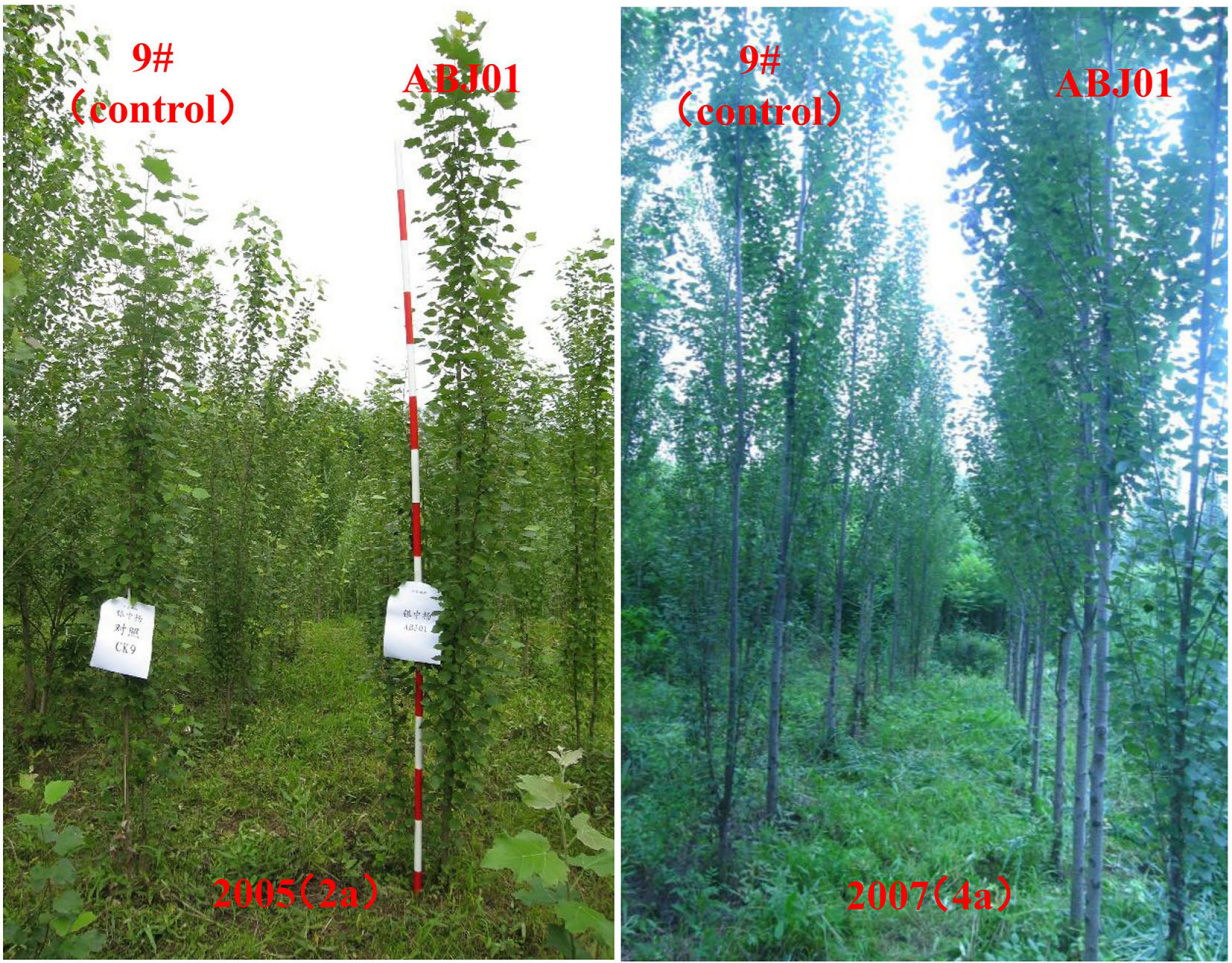
The growth of ABJ01 and 9# in 2005 and 2007 in Panjin. The growth of ABJ01 was significantly faster than 9# in saline soil with 0.25% salt content at Panjin in 2005 and 2007.

Results showed that ABJ01 achieved a larger average height and diameter than 9#in 2004(1a), 2005(2a), 2006(3a), and 2007(4a), respectively (**Figure 1**). The plant height of ABJ01 was 35.60% and 22.94% remarkably higher than 9# in 2006 and 2007, respectively, and the diameter of ABJ01 was 39.31% and 36.32% significantly higher than 9# in 2005 and 2006, respectively, under salt stress conditions (**Figure 1**), which indicated that the salt tolerance of ABJ01 was enhanced compared with 9#. Moreover, the growth of ABJ01 and 9# in Panjin in 2005 and 2007 was shown in **Figure 2**.

**Figure 3:**
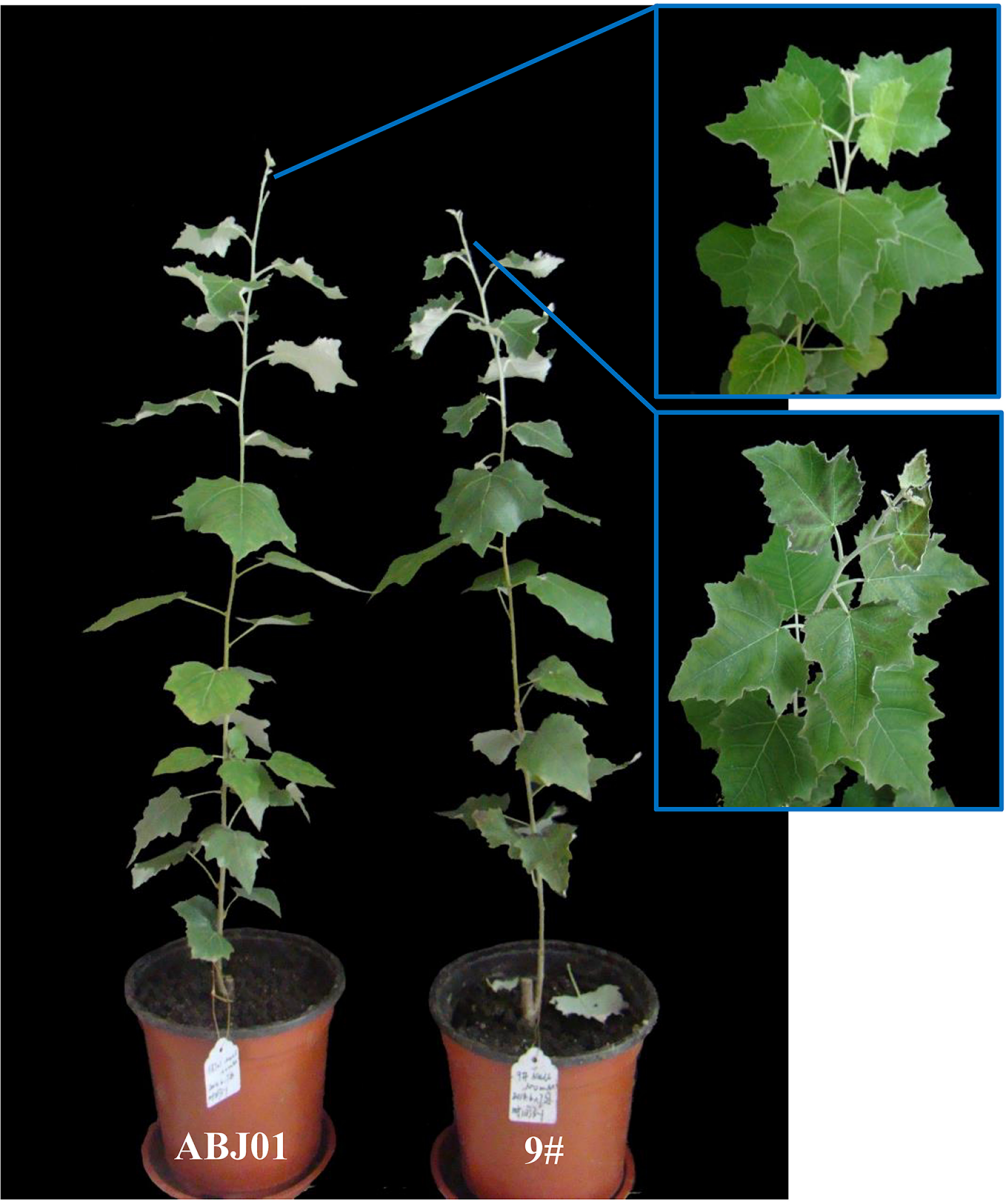
The growth of ABJ01 and 9# under 100mM NaCl stress after 30 days.

For the greenhouse test, after the treatment with 100 mM NaCl for 7 days, some lower leaves of the 9# became yellow and begun to wilt. After 15 days treatment, some leaves had dropped off, the middle and uper leaves had black patches on the surface. After 30 days, more damaged leaves exisited in the middle. Compared to the control, the leaves of ABJ01 showed less wilting at the early stage of NaCl stress, and few lower and middle leaves had yellowed, and no leaves dropped by the end of the experiment (30 days) (**Figure 3**). Under the control condition (0 days) the height of ABJ01 (38.15 cm) was little greater than that of 9# (36.23 cm) with no significant difference. After 100 mM NaCl treatment for 30 days, ABJ01 was significantly higher than 9# with a 4.72% increase (**Table 1**).

**Table 1:** The poplar height with NaCl treatment at 0 day and 30 days.

Clone	0 days(cm)	30 days(cm)
9#	36.23 ± 0.85a	61.93 ± 0.36a
ABJ01	38.15 ± 0.88a	64.85 ± 0.96b

### Expression of *JERF36s*

**Figure 4:**
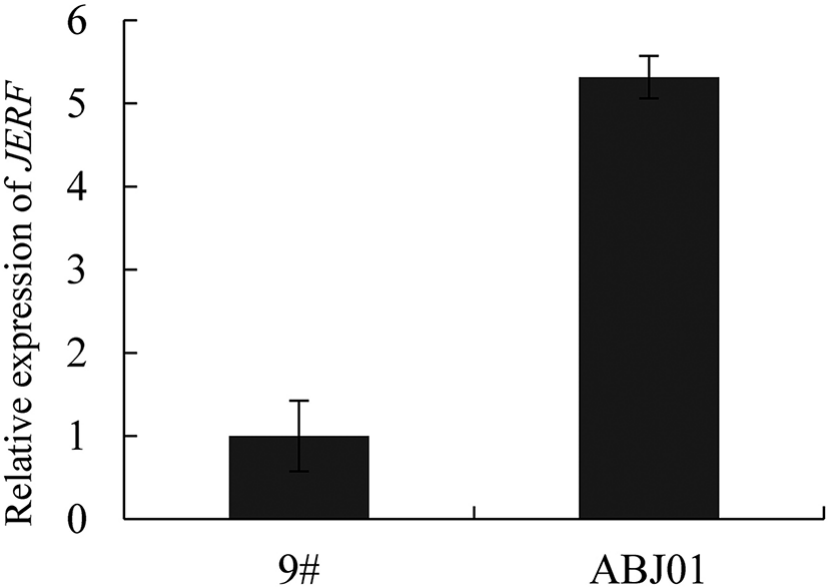
Relative expression of *JERF36s* in 4 years transgenic poplar (ABJ01). Data are given the means and standard errors (error bars) of four repetitions.

To testify the stability of the exogenous gene expression, leaves of 4 years old transgenic popular was used for qRT-PCR analysis. As expected, the expression of *JERF36s* in ABJ01 was significantly higher than 9# (P<0.05), which suggested that it was stably expressed in transgenic line ABJ01 after 4 years (**Figure 4**).

### Expression of *NHX1* and *SOS1* Under Salt Stress

*NHX1* encodes the Na^+^/H^+^ reverse transporter in the vacuole membrane that imports Na^+^ into the vacuole and decreases the Na^+^ concentration in the cytoplasm.

**Figure 5:**
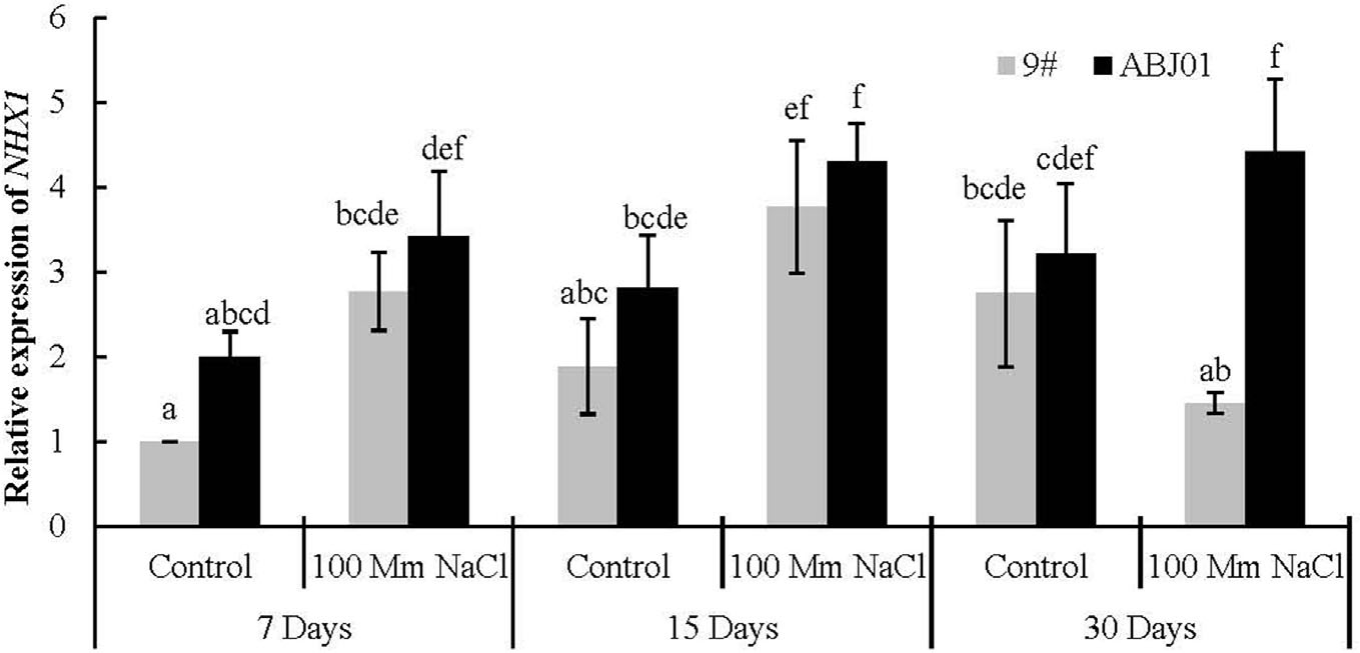
Expression of *NHX1* in 9# and ABJ01 under 100 mM NaCl salt stress and normal water conditions (control) for 7 days, 15 days, and 30 days. The data shows mean ± S.E. of triplicate experiments. Column with different letters indicate significant differences at p < 0.05.

The expression of *NHX1* in ABJ01 was much higher than in 9# under both 100mM NaCl stress and normal water condition (control). And compared with normal condition, ABJ01 and 9# displayed significantly higher expression of *NHX1* under 15 days of salt stress, while the increased expression at 7 days showed no significance. After 30 days stress, the expression of *NHX1* was reduced in 9# compared to control and that in 9# under 15 days treatment, while it was still increase in ABJ01. In addition, the expression of *NHX1* was nearly 4-fold higher in ABJ01 than in 9# under NaCl stress for 30 days. These results suggested that, mild stress can induce the expression of *NHX1* in both ABJ01 and 9#, and with the increase of time, the higher expression of *NHX1* can be effectively maintained in ABJ01 (**Figure 5**).

**Figure 6:**
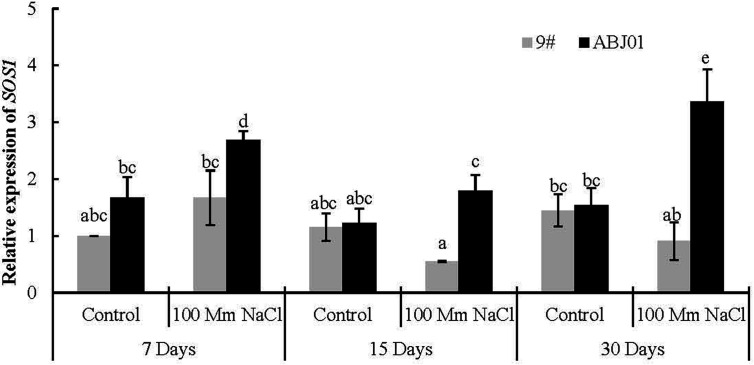
Expression of *SOS1* in 9# and ABJ01 under 100 mM NaCl salt stress and normal water conditions (control) for 7 days, 15 days, and 30 days. The data shows mean ± S.E. of quadruplicate experiments. Column with different letters indicate significant differences at p < 0.05.

In normal water condition, the expression of *SOS1* displayed non-significantly higher in ABJ01 than in 9#. The expression of *SOS1* in ABJ01 changed significantly with first decreased and then increased trend from 7 days to 30 days under 100 mM NaCl salt stress, which was significant higher than in 9# under NaCl stress for 15 days and 30 days, while, the expression of *SOS1* in 9# kept in a low level. In the other hand, the expression of *SOS1* in ABJ01 also significant increased under 7 days and 30 days stressed compared with normal condition, but that was reduced in 9# at days 15 and 30 days. These results suggested that the expression of *SOS1* can be induced in ABJ01 and inhibited in 9# under salt stress (**Figure 6**).

### Na^+^, K^+^, and H^+^ Ion Flux Under Salt Stress

To explore the effects of *NHX1* and *SOS1* expression on ion balance in ABJ01 and 9#, Na^+^, K^+^, and H^+^ ion flux in the apical root tip was measured by the NMT method after the seedlings were treated with 100 mM NaCl for 7, 15, and 30 days.

**Figure 7:**
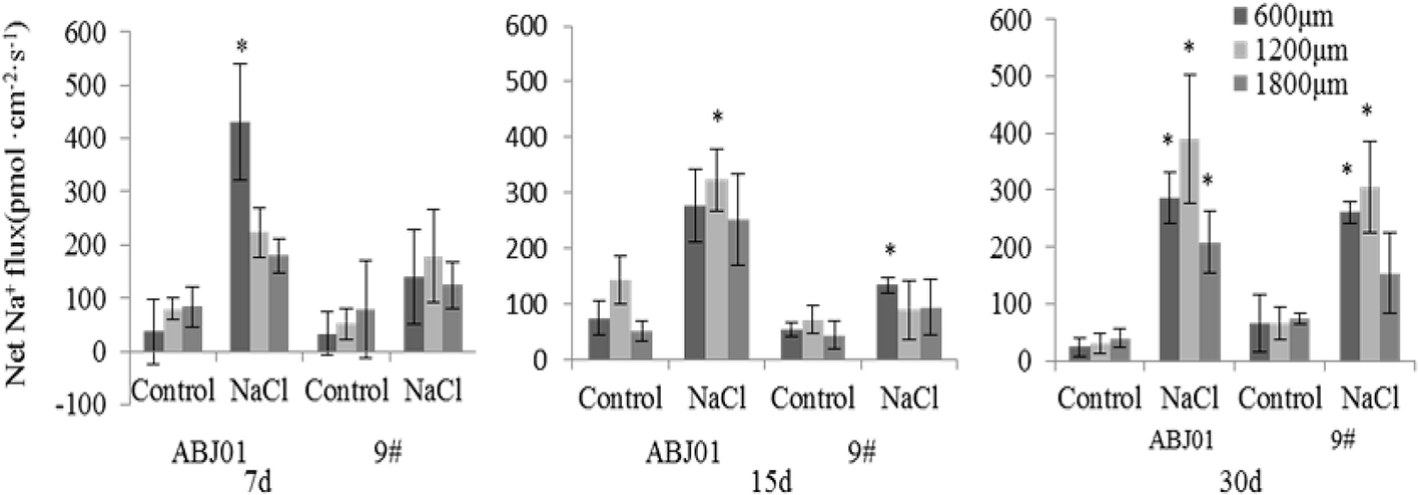
Effects of different salt stress on net Na^+^ flux in the root tip of transgenic (ABJ01) and non-transgenic lines (9#). Positive values indicate efflux, negative values indicate influx; *, significant differences from controls (p < 0.05). Data are given the means and standard errors (error bars) of six repetitions (n = 6).

The results of Na^+^ flux in the apical root tip of ABJ01 and 9# under salt stress and normal condition showed that control groups (normal conditions) for ABJ01 and 9# both displayed lower Na^+^ efflux (<100 pmol cm^-2^ s^-1^) except after 15 days in ABJ01 roots at 1200 μm (Na^+^ efflux >100 pmol cm^-2^ s^-1^). Under salt stress, a higher Na^+^ efflux was observed in both lines. At 7 days, a significant efflux (430 pmol cm^-2^ s^-1^) was observed at the 600 μm root point in ABJ01, but 9# showed no significant differences in Na^+^ efflux. At 15 days of salt stress, a significant Na^+^ efflux (323.53 pmol cm^-2^ s^-1^) was observed at the 1,200 μm point in ABJ01, at the same time, a significant Na^+^ efflux was measured at 600 μm in 9#. At 30 days of salt treatment, Na^+^ efflux was significant at 600, 1200 and 1800 μm in ABJ01 roots. Compared with control, ABJ01 displayed a significantly higher Na^+^ efflux under salt stress, and 9# also exhibited a higher Na^+^ efflux than controls, but a lower efflux than ABJ01. The results therefore showed that Na^+^ efflux was much stronger in ABJ01 than 9# (**Figure 7**).

**Figure 8:**
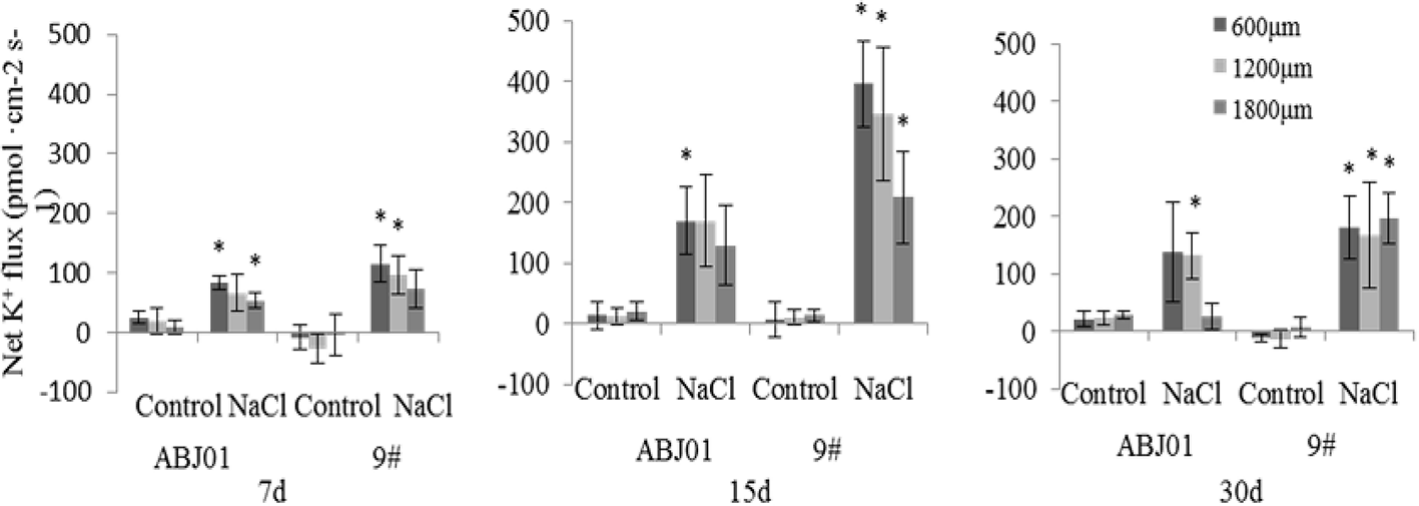
Effects of different salt stress on net K^+^ flux in the root tip of transgenic and non-transgenic lines. Positive values indicate efflux, negative values indicate influx; *, significant differences from controls (*p <* 0.05). Data are given the means and standard errors (error bars) of six repetition (n = 6).

**Figure 9:**
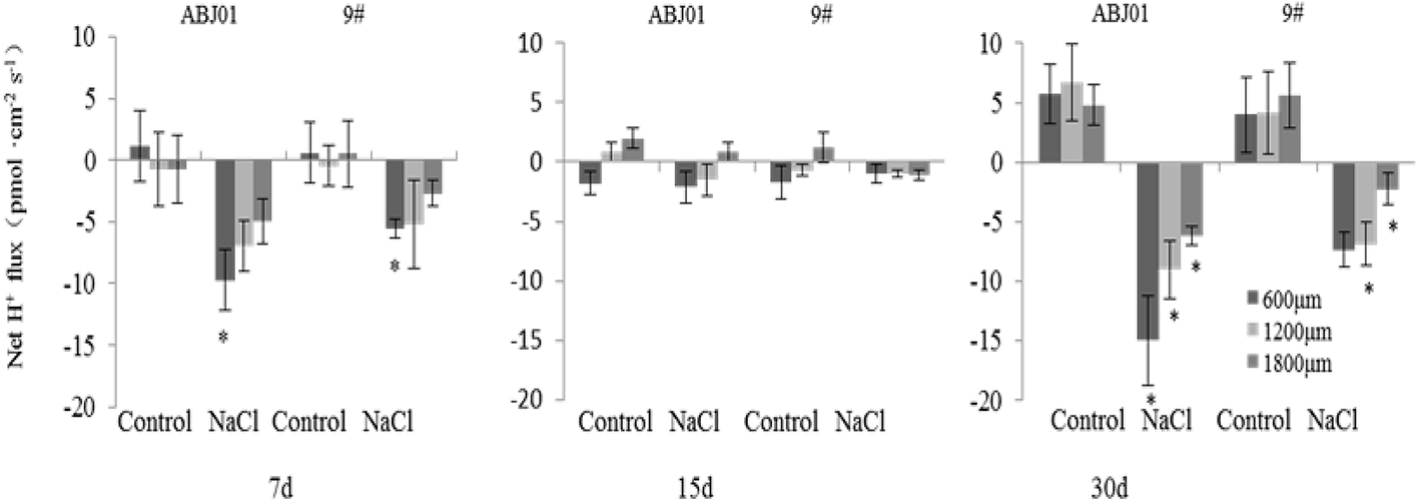
Effects of salt stress on net H^+^ flux in the apical root tip of transgenic and non-transgenic lines. Positive values indicate efflux, negative values indicate influx; *, significant differences from controls (*p <* 0.05). Data are given the means and standard errors (error bars) of six repetition (n = 6).

Under normal condition, net K^+^ efflux showed weakly in ABJ01 at 7, 15, and 30 days, whereas it displayed influx weakly after 7 and 30 days and toward efflux at 15 days in 9#. Under 100mM NaCl salt stress, K^+^ efflux was significant in both ABJ01 and 9#, and the mean K^+^ efflux was highest after 15 days of salt stress in both varieties. After 7 days, ABJ01 showed a significant K^+^ efflux at 600 μm (82.69 pmol cm^-2^ s^-1^) and 1,800 μm (53.66 pmol cm^-2^ s^-1^), while K^+^ efflux in 9# was 115.40 pmol cm^-2^ s^-1^ at 600 μm 96.39 pmol cm^-2^ s^-1^ at 1,200 μm. After salt treatment for 15 days, K^+^ efflux was higher than after 7 days in both ABJ01 and 9#. ABJ01 displayed significant K^+^ efflux at 600 μm (170.53 pmol cm^-2^ s^-1^), and 9# also exhibited significant K^+^ efflux at 600 μm (396.30 pmol cm^-2^ s^-1^), but also at 1200 μm (346.27 pmol cm^-2^ s^-1^) and 1800 μm (209.46 pmol cm^-2^ s^-1^). K^+^ efflux was lower in both ABJ01 and 9# after salt treatment for 30 days, but K^+^ efflux remained significant in 9#. The results showed that K^+^ efflux is much lower in ABJ01 than in 9# under salt stress conditions, suggesting ABJ01 is more capable of maintain Na^+^/K^+^ balance in cells (**Figure 8**). Under normal condition, net H^+^ influx was observed for ABJ01, while H^+^ efflux was observed for 9# after 7 days. After 15 days, H^+^ efflux was observed in ABJ01 and influx was observed in 9#. Both ABJ01 and 9# displayed H^+^ efflux after 30 days. After 7 days salt stress treatment, ABJ01 (-9.73 pmol cm^-2^ s^-1^) and 9# (-5.58 pmol cm^-2^ s^-1^) exhibited significant H^+^ influx at 600 μm. After salt treatment for 15 days, H^+^ influx decreased in both genotypes. At 30 days of NaCl stress, both genotypes exhibited higher H^+^ influx. ABJ01 displayed significant H^+^ influx at 600 μm (-15.00 pmol cm^-2^ s^-1^), 1,200 μm (-9.04 pmol cm^-2^ s^-1^), and 1,800 μm (-6.18 pmol cm^-2^ s^-1^), while 9# displayed significant H^+^ influx at 1,200 μm (-6.89 pmol cm^-2^ s^-1^) and 1,800 μm (-2.26 pmol cm^-2^ s^-1^) (**Figure 9**). All above suggest that ABJ01 is more capable at moving H^+^ through roots than 9#.

## Discussion

It is well known that, maintaining the cellular ion homeostasis by restricting the accumulation of toxic sodium (Na^+^) is the one key response to salt stress, such as a suitable K^+^:Na^+^ ratio to maintain K^+^/Na^+^ balance in the cell, which prevents cellular damage and nutrient deficiency (Clarkson and Hanson, 1980; Tester and Davenport, 2003; Li et al., 2017; Liang et al., 2018; Yang and Guo, 2018; Zhang X. N. et al., 2019). The SOS pathway (exports Na^+^ ions out of cells and is highly conserved in plants) plays an important role in maintaining ion homeostasis, and salt tolerance at cellular in plants (Zhu, 2000; Ji et al., 2013; Yang and Guo, 2018; Cheng et al., 2019; Liu et al., 2019; Mahi et al., 2019; Zhang X. N. et al., 2019). Previous studies have shown that *SOS1*, *SOS2* and *SOS3* are the key components of the SOS signalling pathway involved in Na^+^ extrusion (Zhu et al., 1998; Zhu, 2003; Quintero et al., 2011; Cheng et al., 2019; Foster and Miklavcic, 2019; Zhang X. N. et al., 2019). The Na^+^/H^+^ antiporter responsible for Na^+^ active efflux is encoded by *SOS1*, and proteins encoded by *SOS2* and *SOS3* mainly modulate the activity of SOS1 (Zhu, 2003; Quintero et al., 2011; Fahmideh and Fooladvand, 2018; Foster and Miklavcic, 2019; Yin et al., 2020). And the *SOS1* is the most sensitive gene to different levels of hypersensitivity to NaCl (Wu, 1996; Shi et al., 2000; Yang and Guo, 2018; Yin et al., 2020). Salt tolerance in plants is mediated by Na^+^ extrusion from the cytosol by the plasma membrane Na^+^/H^+^ antiporter *SOS1*, which transports excess Na^+^ from the cytoplasm to the extracellular region (Shi et al., 2000; Cheng et al., 2019; Zhang X. N. et al., 2019; Yin et al., 2020). Overexpression of genes in the SOS pathway, such as *AtSOS1*, can increase salt tolerance in plant, including *Arabidopsis*, *Festuca arundinacea*, *Nicotiana tabacum*, and *Agrostis grass* (Shi et al., 2003; Yue et al., 2012; Ma et al., 2014; Liu et al., 2019). As a primary adaptive mechanism for reducing cytoplasmic ion toxicity in plants grown under high salt concentrations, vacuolar Na^+^ compartmentalization is mediated by the vacuolar Na^+^/H^+^ antiporters (NHX), which are ubiquitous and catalyze the electroneutral exchange of H^+^ for Na^+^ or K^+^ (Barragán et al., 2012; Yang and Guo, 2018; Bassil et al., 2019; Zhang X. N. et al., 2019). The K^+^(Na^+^)/H^+^ antiporter located in the vacuole membrane is encoded by *NHX1* (Venema et al., 2002; Barragán et al., 2012; Zhang X. N. et al., 2019). Plants overexpressing *AtNHX1* have higher vacuolar K^+^ content than Na^+^ even under moderate NaCl stress (Leidi et al., 2010; Li et al., 2017; Yang et al., 2017; Liu et al., 2019). The *NHX1* protein can transport Na^+^ into the vacuole and decreases the Na^+^ concentration in the cytoplasm (Mahajan et al., 2008; Ding et al., 2010; Tang et al., 2010; Yamaguchi et al., 2013; Fahmideh and Fooladvand, 2018; Zhang X. N. et al., 2019). Overexpression of *NHX1* in salt-stressed *Arabidopsis*, *Brassica napus*, *Zea mays*, *Triticum aestivum*, and *A. grass* can improve salt resistance owing to altering the distribution of Na^+^ and K^+^ (Apse et al., 1999; Zhang et al., 2001; Xue et al., 2004; Li et al., 2017; Yang et al., 2017; Liu et al., 2019). In our study, the expression of *NHX1* and *SOS1* were significant higher in ABJ01 than in 9# after 15 days or 30 days salt treatment, and was up-regulated significantly in ABJ01 compared with normal water condition and down-regulated in 9# after 30 days. These results may suggest that the ABJ01 have potential high Na^+^, K^+^/H^+^ antiporter activity and better maintaining intracellular ion homeostasis in the cytoplasm effectively, which greatly reduces the ion toxicity and nutrient deficiency.

Plants minimize K^+^ loss through active efflux of Na^+^ and separation of Na^+^ in the vacuole to maintain K^+^/Na^+^ balance (Zhu, 2003; Chen et al., 2007; Li et al., 2017; Liang et al., 2018; Yang and Guo, 2018; Zhang X. N. et al., 2019). The ability of exporting Na^+^ from cytoplasm to apoplast and partitioning vacuolar Na^+^ into the vacuole becomes a primary adaptive mechanism for reducing cytoplasmic ion toxicity in plants grown under high salt concentration (Hasegawa, 2013; Yang and Guo, 2018; Bassil et al., 2019; Su et al., 2019; Zhang X. N. et al., 2019). And the capability to retain more intracellular K^+^ than Na^+^ helps plants to withstand stress from Na^+^ shock. Electrogenic proton (H^+^) pumps depend on counter-ion fluxes to establish transmembrane pH gradients at the plasma membrane and endomembranes, alkali cation/H^+^ antiporters can alter pH and/or cation homeostasis locally and transientl (Li et al., 2017; Liang et al., 2018; Sze and Chanroj, 2018; Yang and Guo, 2018; Zhao N. et al., 2018; Bassil et al., 2019; Su et al., 2019; Zhang X. N. et al., 2019). Thus, H^+^ is important to maintain the vacuole ion balance for plant under salt stress. Our ion flux results showed that, Na^+^ efflux and H^+^ influx were higher in ABJ01 than in 9# under NaCl stress, consistent with a previous study on *P. euphratica* (Sun et al., 2009a). Other researchers also reported a higher H^+^ influx in wild Arabidopsis grown under salt stress (Shabala, 2000; Shabala et al., 2005). Na^+^ can compete with K^+^ and inhibit K^+^ influx, resulting in an imbalance of the K^+^/Na^+^ ratio that eventually causes ion toxicity in plants (Zhang X. N. et al., 2019). Therefore, restricting the K^+^ efflux is important for the salt resistance of *Populus*. Our results showed that K^+^ efflux was lower in ABJ01 than in 9# under salt stress, indicating less K^+^ loss in ABJ01. This was also the case for *Populus* (Sun et al., 2009b; Zhao N. et al., 2018), *Hordeum vulgare* (Chen et al., 2007), and *T. aestivum* (Cuin et al., 2008). In *H. vulgare* (Chen et al., 2007) and *T. aestivum* (Cuin et al., 2008), the root maturation region was measured at 10,000 μm from the root tip, whereas we measured 600, 1,200, and 1,800 μm from the root tip, which corresponds to the meristematic and elongation zones of the root. *P. euphratica* (Sun et al., 2009b) revealed a change on K^+^ flux in the root tip area (200–2,000 μm) and a smaller change in the meristematic zone (10,000-12,000 μm). Na^+^ efflux and K^+^ influx in roots of two poplar species (*P. euphratica*, *P. popularis*) were recorded in theapical region (ca. 300 μm from the root tip) (Zhao N. et al., 2018). Exogenous expression of Vacuolar membrane-localized *GmNHX1* enhances plant salt tolerance through increasing the efflux rate of Na^+^ in roots and maintaining a high K^+^/Na^+^ ratio along with inducing the expression of Na^+^/H^+^ exchanger *SKOR*, *SOS1*, and *AKT1* (Sun et al., 2019a). All above suggest that higher net Na^+^ efflux, H^+^ influx, and a greater capacity to inhibit K^+^ efflux in ABJ01 can effectively improve the salt tolerance, which may be caused by the higher expression of *NHX1* and *SOS1* in ABJ01.

*JERF36s* belong to the ERF transcription factor class, which are involved in signal transduction, hormone synthesis, and stress responses (Li et al., 2009; Zhang and Huang, 2010; Su et al., 2011). Previous researches have demonstrated that ERF transcription factors could enhance stress resistance in *A. thaliana*, *N. tabacum*, *Lilium longifloruma*, and *Populus* (Gilmour et al., 2000; Wang et al., 2004; Li et al., 2006; Li et al., 2009; Su et al., 2011; Yao et al., 2016; Yao et al., 2017; Charfeddine et al., 2019; Chen et al., 2020; Jiang et al., 2020). Expression of *JERFs* activates the expression of resistance-associated genes and promotes the ability of plants to adapt to environmental stress. And these induced genes in turn may participate in the generation of hormones like ABA (abscisic acid), salicylic acid, and ethylene. In the other hand, these gene products may amplify the initial signal and initiate a second round of signalling that may follow the same pathway or altogether different component of signalling pathway (Basu and Roychoudhury, 2014). For example, *ERF76* (a highly salt-inducible ERF gene) was proved to activate the expression of other transcription factors in the transgenic poplar lines overexpressing *ERF76* under multiple abiotic stresses (Yao et al., 2019). Overexpression of *ERF76* in transgenic poplar upregulated the expression of 16 transcription factor genes and 45 stress-related genes and also increased the ability of ABA and GA (gibberellic acid) biosynthesis, which resulted in stronger tolerance to salt stress (Yao et al., 2016). Overexpression of *PalERF109* increased the transcript abundance of salt stress-related genes, enhanced the ABA signaling pathway, and induced expression of salt many ion channel proteins as well as transporters in transgenic poplars with higher salt tolerance (Chen et al., 2020). Plant hormones and plant hormone signaling pathways regulate complex signaling networks related to plant development and responses to environmental stresses (Bari and Jones, 2009; Zhu, 2016). Under the exogenous ABA treatment, the expression of *SOS-3* and *NHX-1* genes was upregulated in the all rice varieties (Basu and Roychoudhury, 2014). Transgenic lines revealed significantly increased transcript levels of *BjSOS2* gene after ABA treatment (Kaur et al., 2015). In our study, ABJ01 stably overexpressing *JERF*36s grew significantly higher (by more than 20%–30%) and reached a larger diameter than 9# in 2005 and 2006 at Panjin with 0.25% salt content. The higher expression of *NHX1* and *SOS1* in ABJ01 than 9# both was found under both 100 mM NaCl stress and normal condition, especially at 15 days. This may be explained that the overexpression of *JERF36s* in ABJ01 has regulate some signaling networks such as ABA signaling pathway and salt-overly-sensitive (SOS) pathway for responses against environmental stresses, and lead to the increased content of plant growth regulators, such as ABA, gibberellin 3 (GA3), salicylic acid (SA), and jasmonic acid (JA), and then up-regulated the expression of downstream target genes (*NHX1* and *SOS1*) in plant for improving the salt tolerance. Consider of this, up-regulating the expression of genes encoding Na^+^, K^+^/H^+^ antiporters, such as of *NHX1* and *SOS1*, by overexpression of exogenous *JERF36s* may be one of the reason for the improved salt tolerance of transgenic poplars under salt stress.

## Conclusion

In conclusion, ABJ01 exhibited stable expression of *JERF36s*, higher expression of *SOS1* and *NHX1*, stronger Na^+^ efflux and H^+^ influx, and smaller K^+^ loss than 9# in this study, suggesting that exogenous JA/ET responsive transcription factors could regulates Na^+^/H^+^ exchanger *SOS1* and *NHX1* expression, which improve the ability maintain K^+^/Na^+^ homeostasis in root cells for the improved tolerance to salt stress in plants. However, the mechanism by which *JERFs* modulate the expression of *SOS1* and *NHX1* remains to be elucidated and requires further research.

## Data Availability Statement

All datasets generated for this study are included in the article/**Supplementary Material**.

## Author Contributions

Conceptualization: XS and QH. Methodology: WZ, DL, and CD. Formal analysis: WZ and DL. Data curation: DL and CD. Investigation: YD and JL. Writing—original draft preparation: DL and CD. Writing—review and editing: CD and WZ. Supervision: QH. Project administration: CD and WZ. Funding acquisition: XS and CD. All authors contributed to the article and approved the submitted version.

## Funding

This research was funded by the National Major Project of GMO New Species Cultivation (Grant No. 2018ZX08020002) and the National High-Tech R&D Program of China (863 Program) (Grant No. 2011AA100201).

## Conflict of Interest

The authors declare that the research was conducted in the absence of any commercial or financial relationships that could be construed as a potential conflict of interest.
