# Supplementary material for: Effect of Overexpression of JERFs on Intracellular K+/Na+ Balance in Transgenic Poplar (Populus alba × P. berolinensis) Under Salt Stress
Source: Front Plant Sci. 2020 Aug 14;11:1192. doi: 10.3389/fpls.2020.01192 (PMC7456863; doi:10.3389/fpls.2020.01192)
Supplement: Supplementary file 1 [file Table_1.pdf]

SUPPLEMENTARY TABLE S1 | Primers used for qRT-PCR.

Gene	Product length (bp)	Primer sequence
NHX1	150bp	5’-CTTGAGTAGTTAGCAAGATCAAAGT-3’<br>5’-CATCATTCATTACCTTGTTTGTGG-3’
SOS1	306bp	5’-TTGTGGTCTATCAGCTATTCTATCGG-3’<br>5’-CCATCACCCTTGAAAGCAGTCCT-3’
JERF	123bp	5’-CTCTCAACCCCAAACGAGCTC-3’<br>5’-CACGAATTTCAGCAGCCCA-3’
UBQ-like	195bp	5’-TGAGGCTTAGGGGAGGAACT-3’<br>5’-TGTAGTCGCGAGCTGTCTTG-3’
